# Factors related with lung functions among Orang Asli in Tasik Chini, Malaysia: a cross-sectional study

**DOI:** 10.1186/s12889-024-19296-x

**Published:** 2024-07-05

**Authors:** Nur Fadhilah Mohd Razib, Halim Ismail, Roszita Ibrahim, Zaleha Md Isa

**Affiliations:** Department of Public Health Medicine, Faculty of Medicine, National University of Malaysia, Kuala Lumpur, 56000 Malaysia

**Keywords:** Lung functions, Spirometry, Risk factors, Orang Asli, Tasik Chini

## Abstract

**Background:**

Orang Asli lifestyle and household setting may influence their health status especially respiratory system and lung functions. This cross-sectional study was carried out to investigate the status of lung functions of Orang Asli community and the associated factors.

**Methods:**

Data collection was carried out from November 2017 until May 2018 among 211 Orang Asli respondents aged 18 years old and above, who lived in five villages in Tasik Chini, Pahang. All respondents who fulfilled the inclusion criteria were recruited in this study. Interview-guided questionnaire was administered, and spirometry test that include Forced Expiratory Volume in one second (FEV_1)_, Forced Vital Capacity (FVC), and Peak Expiratory Flow Rate (PEFR) was carried out. Data were analyzed using SPSS software version 23.0. In the first stage, descriptive analysis was done to describe the characteristics of the respondents. In the second stage, bivariable analysis was carried out to compare proportions. Finally, multiple logistic regression was performed to assess the effects of various independent predictors on spirometry parameters.

**Results:**

The respondents’ age ranged from 18 to 71 years old in which 50.2% of them were female. The majority ethnicity in Tasik Chini was Jakun tribe (94.3%). More than half of the respondents (52.1%) were current smoker, 5.2% were ex-smoker and 41.7% were non-smoker. More than half of them (62.1%) used woodstove for cooking, compared to only 37.9% used cleaner fuel like Liquefied Petroleum Gas (LPG) as a fuel for everyday cooking activity. The lung function parameters (FEV_1_ and FVC) were lower than the predictive value, whereas the ratio of Forced Expiratory Volume in one second and Forced Vital Capacity (FEV_1_/FVC) (%) and PEFR were within the predictive value. The FEV_1_ levels were significantly associated with age group (18–39 years old) (*p* = 0.002) and presence of woodstove in the house (*p* = 0.004). FVC levels were significantly associated with presence of woodstove in the house (*p* = 0.004), whereas there were no significant associations between all factors and FEV_1_/FVC levels.

**Conclusions:**

FEV_1_ levels were significantly associated with age group 18–39 years old, whereas FVC levels were significantly associated with the presence of woodstove in the house. Thus, environmental interventions such as replacing the use of woodstove with LPG, need to be carried out to prevent further worsening of respiratory health among Orang Asli who lived far from health facilities. Moreover, closer health monitoring is crucial especially among the younger and productive age group.

**Supplementary Information:**

The online version contains supplementary material available at 10.1186/s12889-024-19296-x.

## Background

Poor household indoor air quality is likely to be the biggest concern of public health, especially in countries with resource constrained. Indoor air quality (IAQ) is defined as the air quality within buildings and its structures that has association with the health and well-being of building occupants [1]. Household air pollution’s exposure, discomfort working environment, and fuel type used at household level especially for cooking have been related to respiratory symptoms, thus will also affect their lung function. In recent years, there is more concern related to IAQ by scientists and the public as 70–90% of people spend their daily activity and time indoors such as in the office, workplace, school, and in houses for housewives and people who are not working [2, 3]. Several research such as research by Montgomery & Kalman (1989) [4] and Fairus et al. (2011) [5] reported that indoor air pollution levels surpassed the outdoor pollution levels. From prehistoric times when people migrate from outdoor living to live indoors and later use fire into closed living space for cooking and heating, indoor air pollution has been taking place [6]. As people spend their times 16–18 h in average indoors, indoor air pollution might be more harmful and give rise to more health hazards to the occupants. The data from previous research shows that indoor air pollution located just below malnutrition and poor water quality or sanitation risk-wise [7–10].

There are some difficulties in estimating the reference value for lung function test because it differs in individuals. The interpretation of lung function test mostly used anthropometric factors, including weight, height, sex, and age as basic factors according to clinical guideline [11]. There are other factors or parameters that may associate with the lung function’s estimation. Throughout the reviews on several researches, the factors or parameters includes physical parameters such as circadian rhythms [12], menstrual cycle [13], chest diameter [14], social and health care considerations (educational level) [15], socioeconomic status [16], and workplace exposures [17]; environmental factors such as air pollution [18], climatic conditions [19], and natural disasters [20]; race or ethnic group [21]; lifestyle such as nutrition [22], level of physical activity [23], and smoking [24]; diseases such as diabetes [25], muscle or hormone disorders [26]; physical position [27]; genetic factors [28]; war situations such as military conflicts [29] and terrorist attacks [30]; as well as childhood influencing factors [31] or pregnancy [32]. The review done by Talaminos Barroso et al. (2018) [33] that focus on different factors such as anthropometry, physical position during spirometry was done, race of the respondents and ethnic group shows that all the factors reviewed affect lung function.

Orang Asli is the Malay term for the aborigines or indigenous, and the term literally means ‘original people’. They inhibit the highlands and the peripherals of the hinterlands, hinging on the fringes of the urban area [34]. In Malaysia, a combination of public data from the Department of Orang Asli Development (JAKOA) and data from the Malaysia Statistics Department estimated that the Indigenous Peoples of Malaysia made up 13.7 percent of the 32,382,300 million national population in 2018, including Orang Asli in Peninsular Malaysia and Natives from Sabah and Sarawak [35, 36]. According to the Department of Orang Asli Development (JAKOA) (2021) [36], there were 178,197 Orang Asli in Peninsular Malaysia. Senoi is the largest tribe, accounting for 97,856 (54.9%), followed by Proto Malay 75,332 (42.3%) and the least is Negrito at 5,009 (2.8%) [35]. There are high rates of morbidity and mortality among Orang Asli population. Incidence of tuberculosis in Orang Asli community are three times as the national average. If compared with most of the Malaysian population, Orang Asli was still left behind especially in basic infrastructure, literacy and education, and the poverty rate is quite high. Majority of them still use their traditional living way that pass through their ancestral and make their living by hunting and searching for food in the jungle. As time goes on, there are many changes that recently occur at their settlement such as logging and developers’ encroachment into their land and this condition make some Orang Asli tribes moved away and lived near the cities and towns. This so-called forced urbanisation led to lifestyle and working style changes. Thus, this modification from active daily activities to sedentary lifestyle have direct effect to their lung capacity [37].

Health hazards among Orang Asli have been underestimated in the developing countries. Different tribes of Orang Asli live in different types of housing. Their house structure might be different between each other, and kitchen structure may be different. Some Orang Asli tribes use outdoor kitchen to cook, but some of them who are already modern, use indoor kitchen and gas as fuel. Higher number of populations in the world which count almost three billion continue to focus on using solid fuels known as polluting fuels such as biomass fuels (wood, dung, and agricultural residues), kerosene and coal for their energy sources. Usage of polluting fuels on traditional stove or fire for cooking and heating will produce very high levels of household air pollution that contains a vast range of dangerous pollutants (small particles, carbon monoxide and particulate matter) that cause various health problems. The levels may reach 20 times higher than accepted guideline values [38]. Various health outcomes may develop due to exposure to household air pollution such as ischaemic heart disease, stroke, chronic obstructive pulmonary disease (COPD) and lung cancer in adults. For children, the most common health effect is acute lower respiratory infections. According to WHO (2016) [38], 7.7% of global mortality is contributed by household air pollution.

It is presumed that the modernised Orang Asli settlement in Malaysia is a community of indigenous population in the state of transition from the traditional health paradigm to the modern one. Their lifestyle and household setting such as modern or traditional types of houses may influence their health especially their respiratory system. They might not think that indoor air quality plays a role in their health. Thus, this study will be carried out to investigate the lung functions among Orang Asli community and the factors associated with indoor air quality that may affect their lung functions.

There are very limited studies done locally among the indigenous community with regards to respiratory symptoms and lung functions and their relationship with household factors. This is the first study to measure the lung functions of the indigenous community focusing on the Orang Asli in Tasik Chini settlement in Pahang, where they have different household settings between five Orang Asli villages in Tasik Chini. Lung functions might be a very useful indicator to determine its association with indoor air quality in different household areas of different groups of population. Comparison will be made according to age group, types of houses, housing area such as rural, urban, or industrialised area and others. There were several studies done to investigate the lung functions of population, but they were focusing on urban and rural population only and mainly focus on outdoor air pollution but not among the Orang Asli. Thus, the choice on the scope of the study was a perfect fit as there are limited literature on the topic. Therefore, the main objective of this study was to determine the association between indoor air quality and lung functions among Orang Asli community in Tasik Chini, Pahang. Meanwhile, the specific objectives of this study are to i) determine the status of lung function of Orang Asli community in Tasik Chini; ii) identify the type of housing and lifestyle of Orang Asli community in Tasik Chini that may influence their lung function status; and iii) assess the relationship between indoor environmental exposure (smoke from ETS and woodstove, home environment and dust in the house factor) and lung function status among Orang Asli community in Tasik Chini. It is hypothesized that there is a relationship between the type of housing and lifestyle and indoor environmental exposure and the lung function status of Orang Asli community in Tasik Chini.

## Methods

### Setting and location

This study was conducted among Orang Asli community in Tasik Chini, Pahang. Tasik Chini is in the southeastern state of Pahang, which is about 100 km from Kuantan and 60 km from the town Pekan. There are five villages where the Orang Asli lived near the freshwater lake. The villages are Kampung Gumum (main village), Kampung Cendahan, Kampung Tanjung Puput, Kampung Melai and Kampung Ulu Gumum. Most Orang Asli around the lake is from the Jakun tribe.

Most of the Orang Asli in Kampung Gumum and Kampung Ulu Gumum lived in modern houses located near town. This is contrary to the people living in Kampung Cendahan, Tanjung Puput and Melai which are located near the lake and situated deeper inside the forest, in which majority of them lived together as family in their traditional houses that is connected to the indoor kitchen without any partition between living room, bedroom and kitchen. Some of them used outdoor kitchens for cooking and used wood as a type of fuel for cooking. Thus, higher accumulation of indoor air pollutants may affect their respiratory conditions and may worsen their health.

### Study design

**Fig. 1:**
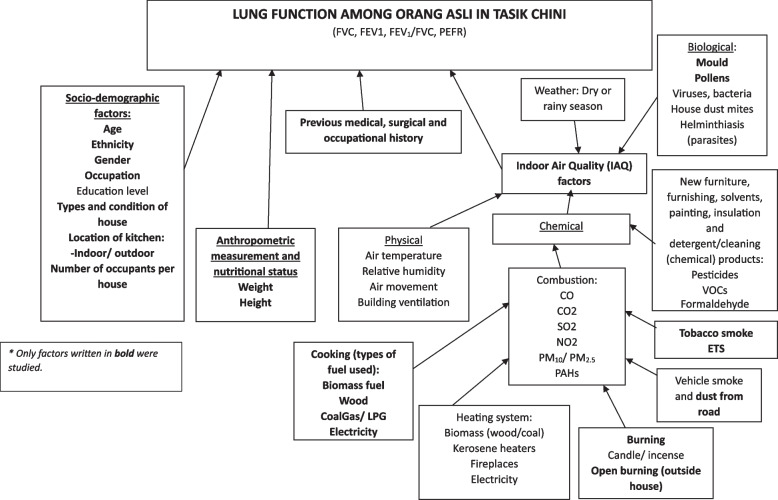
Conceptual framework

A cross-sectional study design was used to determine the lung functions of Orang Asli in Tasik Chini. The associations of respiratory symptoms and lung functions with sociodemographic factors, housing area, types of houses, kitchen location, number of occupants per house, tobacco exposure and cooking and heating source were determined in this study. Figure 1 shows the conceptual framework of the study.

### Sample size

The sample size is determined by referring to a table for determining sample size for finite population derived from Krejcie & Morgan (1970) [39]. Based on the number of Orang Asli in Tasik Chini gathered from the Department of Orang Asli (JAKOA), the sample size required for this study was 127.

### Data collection

All adult villagers were screened and a total of 211 respondents who fulfilled the inclusion criteria were recruited from November 2017 until May 2018. The inclusion criteria were age 18 years old and above, had lived in the housing area for at least 1 year, and no previous history of hospital admission due to chronic respiratory and cardiovascular problem and abdominal, cardiovascular and eye operation in the last six months prior to data collection. The exclusion criteria were tuberculosis patients or respondents with history of tuberculosis, respondents who were not at home during the time of data collection, and respondents who refused to be enrolled in the study.

### Study instruments

#### Interview-guided questionnaire

The questionnaire was adapted from a study entitled `Indoor environmental factors associated with asthma and asthma-related symptoms among adults: a comparison between summer and winter in Zunyi China’ [40]. The questionnaire was based on the adult questionnaire of the European Community Respiratory Health Survey II (ECRHS II). It contained standardized questions on doctors diagnosed asthma, current asthma, and other asthma-related symptoms, as well as household environmental factors. The adapted and modified questionnaire went through forward and backward translation from English to Malay and back to English by qualified English-Malay teachers. Both original and newly translated English versions were compared in term of meaning before the Malay version questionnaire was finalized. The value of Cronbach alpha was 0.7 after the reliability test was done. The questionnaire is divided into four main sections that includes sociodemographic factors (age, occupation, ethnicity, and housing area); history of tobacco smoke exposure (health status, family history and tobacco smoke exposure); home environment; and dust and air pollution inside the house.

### Lung function test

The lung function test was performed by a trained medical officer using MicroDirect MicroGP Spirometer that has been calibrated before use. Lung function forced expiratory volume in 1 s (FEV_1_), forced vital capacity (FVC), FEV_1_/FVC, and peak expiratory flow rate (PEFR) were measured using standardized procedures and acceptability criteria was recommended by the American Thoracic Society. Lung function values were predicted based on age, height, weight, and body mass index (BMI). Each spirometer was calibrated at the beginning of the survey and checked periodically in series. At least three satisfactory blows were recorded. The subject's technical performance was assessed by the researchers and reading which is considered technically unsatisfactory was discarded. All data received were entered into the Global Lung Function Initiative (GLI) network system and the predicted value for each parameter generated by the system was compared with the result from respondents to determine the status of the lung function. The GLI network has produced standardized lung function reference values for spirometry and gas transfer tests. Table 1 shows the operational definition of variables.

**Table 1:** Operational definition of variables

Item number	Variable	Operational definition
Dependent variable		
1	Forced expiratory volume in 1 s (FEV1)	Normal: ≥ 80% predicted normal; Obstructive: < 80% predicted normal; Restrictive: < 80% predicted normal; Combined: < 80% predicted normal
2	Forced vital capacity (FVC)	Normal: ≥ 80% predicted normal; Obstructive: < 80% predicted normal (reduced but to a lesser extent than FEV1); Restrictive: < 80% predicted normal; Combined: < 80% predicted normal
3	The proportion of the forced vital capacity exhaled in the first second (FEV1/FVC)	Normal: > 0.7; Obstructive: < 0.7; Restrictive: > 0.7; Combined: < 0.7
4	Peak expiratory flow rate (PEFR)	Peak expiratory flow rate (PEFR) is the maximum flow rate (expressed in liters per minute [L/min]) generated during a forceful exhalation, starting from full inspiration. Green zone: 80 to 100 percent of your usual or "normal" peak flow rate signals all clear Yellow zone: 50 to 80 percent of your usual or "normal" peak flow rate signals caution Red zone: Less than 50 percent of your usual or "normal" peak flow rate signals a medical alert
Independent variable		
5	Age	Age was categorized into three groups (18–39, 40–59 and > 60 years old) which represent young, middle and old age of the respondents
6	Gender	Gender as stated in the identification card, either male or female
7	Ethnicity	Ethnicity as stated on the identification card. The ethnicity includes Jakun tribe, Semoq Beri and Semelai tribes, Malay, Chinese and Indian
8	Religion	Religion as stated on the identification card. The religion includes Islam, Christian, Buddha and animist, who are without religion
9	Marital status	Marital status of the respondent was divided into two groups: married and not married. Not married includes single, widowed and separated/divorced
10	Occupation	Occupation is defined as the activity that the respondents carried out to make a living and also includes the respondents who only stay at home due to unable or not fit to do any activities that require their strength. The occupation include rubber tapping, gardener/farmer, self-employed or open small business, housewife and others that includes store helper, students and respondents who are not working
11	Body mass index (BMI)	BMI was defined as the individual’s body weight (kilogram, kg) divided by respondent’s height (square meter, m^2^). The respondents’ BMI was categorized into underweight (BMI < 18.5 kg/m^2^), normal weight (18.5 < BMI < 23.0 kg/m^2^) and overweight (BMI > 23.0 kg/m^2^)
12	Type and condition of houses	The type of the house that the respondents currently lived (modern/traditional house, house without bedroom, open kitchen), and the condition of the house whether there is presence of adequate ventilation or not
13	Health status score	Health status factors include previous medical treatment due to respiratory problem, history of childhood asthma and family history of asthma (questionnaire part A; number 1, 1.2, 1.2.1, 1.2.2, 1.2.3, 2, 3, 5, 8 and 9). The median score of health status is the numerical value separating the higher half of the health status score from the lower half. The exposure value was assigned for each source from the questionnaire. The sum of maximum exposure value for health status factor was 23 and the sum of minimum exposure value was 10. The score from 10 to 19 was considered as poor health status and the score from 20 to 23 was considered as good health status
14	Smoke exposure score	Smoke exposure factor includes smoking habit and smoke exposure to ETS and woodstove (questionnaire part A; number 4, 6, 7, 10, 11 and 12). The median score of smoke exposure is the numerical value separating the higher half of the smoke exposure score from the lower half. The exposure value was assigned for each source from the questionnaire. The sum of maximum exposure value for health status factor was 6 and the sum of minimum exposure value was 14. The score from 6 to 9 was considered as more exposed and the score from 10 to 13 was considered as less exposed
15	Home environmental score	Home environmental factors include condition of the house, usage of woodstove in the house, ventilation, and presence of damp and mold area in the house (questionnaire part B; number 1, 1.1, 2, 3, 4, 5, 6, 7, 7.1, 8 and 9). The median score of home environmental factor is the numerical value separating the higher half of the home environmental score from the lower half. The exposure value was assigned for each source from the questionnaire. The sum of maximum exposure value for home environmental factor was 30 and the sum of minimum exposure value was 18. The score from 18 to 23 was considered as poor home environment and the score from 24 to 30 was considered as good home environment
16	Dust in the house score	Dust in the house factor includes presence of dust or pollen inside the house that can be obtained from long term usage of carpet and mattress, keeping pets with fur and feather in the house and bedroom, and dust acquired from outside the house when windows are opened (questionnaire part C; number 1, 2, 4, 5, 5.1, 6 and 7). The median score of dust in the house factor is the numerical value separating the higher half of the dust in the house score from the lower half. The exposure value was assigned for each source from the questionnaire. The sum of maximum exposure value for dust in the house factor was 22 and the sum of minimum exposure value was 7. The score from 7 to 15 was considered as poor environment and the score from 16 to 22 was considered as good environment
17	Type of smoker	a) Non-smoker: respondent who had never smoked for more than 6 months continuous period b) Ex-smoker: respondent who had stopped smoking at least 1 year before this research was conducted c) Current smoker: respondent wo had smoked at least 100 cigarettes (5 packs) in their lifetime and who smoked on daily basis within the time when this research was conducted (King et al. 2010)

### Data analysis

Data collected was analyzed using descriptive and analytical analysis. Descriptive analysis was done to describe the respondents’ characteristics using frequency and percentages for each qualitative data such as ethnicity, occupation, housing area, marital status, while mean and standard deviation were used for quantitative data such as age of the respondents. Subsequently, the bivariable analysis was done by using a chi-squared test to compare proportions and to determine associations between independent and dependent variables.

Non-parametric test analysis was used to estimate the difference in lung function levels (FEV_1_, FVC, FEV_1_/FVC and PEFR) among respondents who were exposed to various indoor exposure factors (health status, smoke exposure, home environment and dust in the house score). Non-parametric analysis was carried out in view of the lung function data not normally distributed.

In the final stage, the significant factors from the bivariable analysis were further tested using multiple logistic regressions and later, the factors that predict the lung functions (FEV_1_, FVC, FEV_1_/FVC and PEFR) among respondents were constructed. Data were analyzed using SPSS software version 23.0. The level of significance was set at 95% with p value < 0.05 for two tailed analyses.

## Results

### Profile of respondents

In this study, a total of 215 respondents from five villages in Tasik Chini, Pahang participated. A total of 4 respondents were excluded from this study due to inadequate information and unable to perform spirometry because of health problem. Overall, 48 families took part in the present study covering 211 people aged 18 years old and above. All respondents answered the questionnaires given to them and performed acceptable spirometry. Table 2 shows the sociodemographic, house, and smoking characteristics of the respondents.

**Table 2:** Sociodemographic, house, and smoking characteristics of the respondents

Characteristics	Frequency (*N* = 211)	Percentage (%)
Age (years) (mean + sd)	35.07 + 12.84	
Age distribution (year)		
18-39	147	69.7
40-59	49	23.2
> 60	15	7.1
Gender		
Male	105	49.8
Female	106	50.2
Ethnic group		
Jakun	199	94.3
Malay	5	2.4
Chinese	3	1.4
Indian	1	0.5
Semoq Beri	2	0.9
Semelai	1	0.5
Religion		
No religion (animist)	169	80.1
Islam	34	16.1
Christian	8	3.8
Marital Status		
Married	193	91.5
Not married	18	8.5
Occupation		
Rubber tapper	66	31.3
Gardener/ Farmer	53	25.1
Self-employed/ small business	43	20.4
Housewife	38	18.0
Store helper	1	0.5
Student	1	0.5
Not working	9	4.3
BMI (kg/m^2^)^a^		
Underweight (BMI < 18.5)	7	3.3
Normal weight (18.5 < BMI < 23.0)	177	83.9
Overweight (BMI > 23.0)	27	12.8
History of asthma in childhood		
Yes	2	0.9
No	209	99.1
Family history of asthma		
Yes	5	2.4
No	206	97.6
Type of house		
Traditional house	94	44.5
Modern house	117	55.5
Bedroom in house		
House with bedrooms	164	77.7
House without bedrooms	47	22.3
**Kitchen location**		
Connected with house	190	90.0
Separate from house	21	10.0
Presence of woodstove in the house		
Yes	162	76.0
No	49	23.0
Types of fuel frequently used for cooking		
Woodstove	131	62.1
Liquefied Petroleum Gas (LPG)	80	37.9
Exposure to smoke from woodstove		
Exposed	162	76.8
Not exposed	49	23.2
Frequency of smoke from woodstove enters the house		
4-5 times per day or more	7	3.3
2-3 times per day	68	32.2
Once per day	28	13.3
2-3 times per week	19	9.0
Rare	40	19.0
Never	49	23.2
History of smoking		
Yes	121	57.3
No	90	42.7
Type of smoker		
Current smoker	110	52.1
Ex-smoker	11	5.2
Non-smoker	90	42.7
Exposure to tobacco smoke from other smoker		
Exposed	204	96.7
Not exposed	7	3.3
Smoking inside house		
Yes	34	16.1
No	101	47.9

^a^BMI cutoffs for Asian and Pacific populations (WHO/IASO/IOTF 2000; James et al. 2002)

Based on the sociodemographic characteristics of the respondents in this study (Table 2), the mean age was 35.07 ± 12.84 years old and 50.2% of the respondents were female. The majority ethnicity in Tasik Chini area was Jakun which constituted the highest proportion (94.3%) compared to other ethnic groups. Majority of the population (91.5%) were married. Most of them worked as rubber tapper (31.3%) and farmer (25.1%) who have their own estate and farm. Majority respondents (83.9%) were having normal weight, 3.3% were underweight, and 12.8% were overweight. None of the respondents were obese. Majority respondents (99.1%) had no history of asthma during childhood and majority (97.6%) had no family history of asthma.

The type of house that the respondents lived and the type of fuel they used for cooking and exposure to the smoke from woodstove usage are shown in Table 2. Majority of the respondents lived in modern houses (55.5%) compared to only 44.5% of them lived in the traditional type of houses. More than three quarters of them lived in the house that was built with bedroom (77.7%) and a quarter of them lived in the house without bedroom (22.3%). Almost all households have kitchen connected to their house (90.0%). A total of 76.0% of respondents have woodstove in their house, and more than half of them used woodstove for cooking. A total of 76.8% of the respondents were exposed to smoke from woodstove since age 5 years old. Due to the kitchen connected to the house, there is a high probability that the smoke from woodstove during cooking will enter the house, but it all depends on how frequent cooking activity is carried out every day.

Meanwhile, more than half of the respondents (57.3%) were smoker. More than half of the respondents (52.1%) still smoke till the day the data was collected (current smoker), 5.2% were ex-smoker and 42.7% were non-smoker. Most of the respondents were exposed to tobacco smoke from other smoker every day. Only 16.1% of the smokers smoked inside their house, whereas 47.9% will go outside of their house to smoke.

### Lung functions (FEV_1_, FVC, FEV_1_/FVC and PEFR) and its associated factors

Table 3 and Table 4 show the lung function status and its association with sociodemographic characteristics, health status, home environment, dust, and smoke exposure (FEV_1_, FVC, FEV_1_/FVC and PEFR) among respondents. There are significant associations between Forced Vital Capacity (FVC) among respondents with type of house (traditional and modern) and presence of woodstove in their house. The respondents who lived in the modern house and have woodstove in their house have abnormal FVC level compared to the respondents who lived in the traditional type of house and who did not have woodstove in their house.

**Table 3:** The parameters of lung functions among respondents (*N* = 211)

	Mean Median	Standard Deviation IQR	Minimum	Maximum	Percentiles		
					25th	50th (Median)	75th
FEV_1_	1.85	0.49	1.10	3.00	1.53	1.63	2.10
FVC	1.96	0.52	1.13	3.60	1.62	1.70	2.27
FEV_1_/ FVC (%)	94.71	5.60	68	100	93	96	98
PEFR	410.35	90.94	201	622	357	393	480

**Table 4:** FEV_1_, FVC, FEV_1_/FVC, PEFR and their association with sociodemographic characteristics, home environment, dust, smoke exposure, and health status among respondents

Variable	Category	FEV_1_		Χ^2^	*p*-value	FVC		Χ^2^	*p*-value	FEV_1_/FVC		Χ^2^	*p*-value	PEFR		Χ^2^	*p*-value
		Normal (*n* = 65)	Abnormal (*n* = 146)			Normal (*n* = 36)	Abnormal (*n* = 175)			Normal (*n* = 209)	Abnormal (*n* = 2)			Normal (*n* = 160)	Abnormal (*n* = 51)		
Age (year)	18–39	36 (24.5)	111 (75.5)	11.052	0.004*	22 (15.0)	125 (85.0)	3.322	0.190	145 (98.6)	2 (1.4)	0.879	0.644	111 (75.5)	36 (24.5)	1.074	0.585
	40–59	20 (40.8)	29 (59.2)			9 (18.4)	40 (61.6)			49 (100.0)	0 (0)			39 (79.6)	10 (20.4)		
	≥ 60	9 (60.0)	6 (40.0)			5 (33.3)	10 (66.7)			15 (100.0)	0 (0)			10 (66.7)	5 (33.3)		
Gender	Male	34 (32.4)	71 (67.6)	0.243	0.622	19 (18.1)	86 (81.9)	0.158	0.691	103 (98.1)	2 (1.9)	2.038	0.153	75 (71.4)	30 (28.6)	2.209	0.137
	Female	31 (29.2)	75 (70.8)			17 (16.0)	89 (84.0)			106 (100.0)	0 (0)			85 (80.2)	21 (19.8)		
Ethnic	Jakun	58 (29.1)	141 (70.9)	4.523	0.033*	31 (15.6)	168 (84.4)	5.444	0.020*	197 (99.0)	2 (1.0)	0.122	0.727	150 (75.4)	49 (24.6)	0.391	0.532
	Others	7 (58.3)	5 (41.7)			5 (41.7)	7 (58.3)			12 (100.0)	0 (0)			10 (83.3)	2 (16.7)		
Religion	No religion (animist)	46 (27.2)	123 (72.8)	5.124	0.024*	24 (14.2)	145 (85.8)	4.909	0.027*	169 (100.0)	0 (0)	8.125	0.004*	126 (74.6)	43 (25.4)	0.751	0.386
	With religion	19 (45.2)	23 (54.8)			12 (28.6)	30 (71.4)			40 (95.2)	2 (4.8)			34 (81.0)	8 (19.0)		
Marital status	Married	58 (30.1)	135 (69.9)	0.603	0.437	31 (16.1)	162 (83.9)	1.597	0.206	191 (99.0)	2 (1.0)	0.188	0.664	147 (76.2)	46 (23.8)	0.140	0.709
	Not married	7 (38.9)	11 (61.1)			5 (27.8)	13 (72.2)			18 (100.0)	0 (0)			13 (72.2)	5 (27.8)		
Occupation	Rubber tapper	16 (24.2)	50 (75.8)	3.768	0.438	9 (13.6)	57 (86.4)	1.856	0.762	64 (97.0)	2 (3.0)	4.436	0.350	47 (71.2)	19 (28.8)	1.663	0.797
	Gardener/Farmer	19 (35.8)	34 (64.2)			9 (17.0)	44 (83.0)			53 (100.0)	0 (0)			43 (81.1)	10 (18.9)		
	Self-employed/ small business	15 (34.9)	28 (65.1)			9 (20.9)	34 (79.1)			43 (100.0)	0 (0)			33 (76.7)	10 (23.3)		
	Housewife	10 (26.3)	28 (73.7)			6 (15.8)	32 (84.2)			38 (100.0)	0 (0)			29 (76.3)	9 (23.7)		
	Others	5 (45.5)	6 (54.5)			3 (27.3)	8 (72.7)			1 (100.0)	0 (0)			8 (72.7)	3 (27.3)		
BMI	Underweight	2 (28.6)	5 (71.4)	0.570	0.752	1 (14.3)	8 (85.7)	0.604	0.739	7 (100.0)	0 (0)	2.527	0.283	5 (71.4)	2 (28.6)	0.138	0.933
	Normal	53 (29.9)	124 (70.1)			29 (16.4)	148 (83.6)			176 (99.4)	1 (0.6)			135 (76.3)	42 (23.7)		
	Overweight	10 (37.0)	17 (63.0)			6 (22.2)	175 (77.8)			26 (96.3)	1 (3.7)			20 (74.1)	7 (25.9)		
History of asthma in childhood	Yes	0 (0)	3 (100.0)	1.355	0.554	0 (0)	3 (100.0)	0.626	1.000	3 (100.0)	0 (0)	0.029	0.864	2 (66.7)	1 (33.3)	0.139	0.566
	No	65 (31.2)	143 (68.8)			36 (17.3)	172 (82.7)			206 (99.0)	2 (1.0)			158 (76.0)	50 (24.0)		
Family history of asthma	Yes	3 (60.0)	2 (40.0)	2.048	0.171	1 (20.0)	4 (80.0)	0.031	0.860	5 (100.0)	0 (0)	0.049	0.825	4 (80.0)	1 (20.0)	0.049	0.826
	No	62 (30.1)	144 (69.9)			35 (17.0)	171 (83.0)			204 (99.0)	2 (1.0)			156 (75.7)	50 (24.3)		
Type of house	Traditional	23 (24.5)	71 (75.5)	3.194	0.074	10 (10.6)	84 (89.4)	4.943	0.026*	94 (100.0)	0 (0)	1.622	0.203	73 (77.7)	21 (22.3)	0.310	0.578
	Modern	42 (35.9)	75 (64.1)			26 (22.2)	91 (77.8)			115 (98.3)	2 (1.7)			87 (74.4)	30 (25.6)		
Bedroom in the house	House with bedrooms	53 (32.3)	111 (67.7)	0.789	0.374	31 (18.9)	133 (81.1)	1.763	0.184	162 (98.8)	2 (1.2)	0.579	0.447	123 (75.0)	41 (25.0)	0.276	0.599
	House without bedrooms	12 (25.5)	35 (74.5)			5 (10.6)	42 (89.4)			47 (100.0)	0 (0)			37 (78.7)	10 (21.3)		
Kitchen location	Connected with house	61 (32.1)	129 (67.9)	1.513	0.219	34 (17.9)	156 (82.1)	0.936	0.337	188 (98.9)	2 (1.1)	0.223	0.637	143 (75.3)	47 (24.7)	0.334	0.563
	Separate from house	4 (19.0)	17 (81.0)			2 (9.5)	19 (90.5)			21 (100.0)	0 (0)			17 (81.0)	4 (19.0)		
Fuel used for cooking	Woodstove	34 (26.0)	97 (74.0)	3.815	0.051	19 (14.5)	112 (85.5)	1.597	0.206	131 (100.0)	0 (0)	3.306	0.069	98 (74.8)	33 (25.2)	0.196	0.658
	Liquefied Petroleum Gas (LPG)	31 (38.8)	49 (61.2)			17 (21.3)	63 (78.8)			78 (97.5)	2 (2.5)			62 (77.5)	18 (22.5)		
Presence of woodstove in the house	Yes	43 (26.5)	119 (73.5)	5.946	0.015*	22 (13.6)	140 (86.4)	5.975	0.015*	162 (100.0)	0 (0)	6.676	0.010*	121 (74.7)	41 (25.3)	0.493	0.483
	No	22 (44.9)	27 (55.1)			14 (28.6)	35 (71.4)			47 (95.9)	2 (4.1)			39 (79.6)	10 (20.4)		
Exposure to smoke from woodstove	Exposed	61 (30.3)	140 (69.7)	0.416	0.519	33 (16.4)	168 (83.8)	1.242	0.265	199 (99.0)	2 (1.0)	0.100	0.751	150 (74.6)	51 (25.4)	3.346	0.067
	Not exposed	4 (40.0)	6 (60.0)			3 (30.0)	7 (70.0)			10 (100.0)	0 (0)			10 (100.0)	0 (0)		
History of smoking	Yes	39 (31.7)	84 (68.3)	0.112	0.737	23 (18.7)	98 (81.3)	0.559	0.455	121 (98.4)	2 (1.6)	1.445	0.229	88 (71.5)	35 (28.5)	2.954	0.086
	No	26 (29.5)	62 (70.5)			13 (14.8)	75 (85.2)			88 (100.0)	0 (0)			72 (81.8)	16 (18.2)		
Type of smoker	Non-smoker	26 (29.5)	62 (70.5)	3.068	0.216	13 (14.8)	75 (85.2)	1.186	0.553	88 (100.0)	0 (0)	1.785	0.410	72 (81.8)	17 (18.2)	3.650	0.161
	Ex-smoker	6 (54.5)	5 (45.5)			3 (27.3)	8 (72.7)			11 (100.0)	0 (0)			9 (81.8)	2 (8.2)		
	Current smoker	33 (29.5)	79 (70.5)			20 (17.9)	92 (82.1)			110 (98.2)	2 (0.9)			79 (70.5)	33 (29.5)		
Exposure to tobacco smoke from other smokers	Exposed	62 (30.4)	142 (69.6)	0.493	0.482	34 (16.7)	170 (83.3)	0.678	0.410	202 (99.0)	2 (1.0)	0.069	0.792	153 (75.0)	51 (25.0)	2.308	0.129
	Not exposed	3 (42.9)	4 (57.1)			2 (28.6)	5 (71.4)			7 (100.0)	0 (0)			7 (100.0)	0 (0)		
Smoking inside house	Yes	14 (41.2)	20 (58.8)	3.222	0.073	7 (20.6)	27 (79.4)	0.248	0.618	34 (100.0)	0 (0)	0.888	0.346	28 (82.4)	6 (17.6)	3.280	0.070
	No	19 (24.4)	59 (75.6)			13 (16.7)	65 (83.3)			76 (97.4)	2 (2.6)			51 (65.4)	27 (34.6)		

*significant at *p* < 0.05

FEV_1_ Forced expiratory volume in one second, FVC Forced vital capacity, FEV_1_/FVCRatio of forced expiratory volume in one second (FEV_1_) and Forced vital capacity (FVC), Peak expiratory flow rate (PEFR)

Predictors of the lung functions (FEV_1_, FVC, FEV_1_/FVC and PEFR) of the respondents.

Multiple logistic regression analysis was performed to assess the predictors of the lung functions (FEV_1_, FVC and FEV_1_/FVC). Table 5 showed that FEV_1_ levels were significantly associated with age group (18–39 years old) (*p* = 0.002) and presence of woodstove in the house (*p* = 0.004). FVC levels were significantly associated with the presence of woodstove in the house (*p* = 0.004) as shown in Table 6. There is no significant association between all factors studied and FEV_1_/FVC levels (Table 7).

**Table 5:** Predictors of the Forced Expiratory Volume in one second (FEV_1_) among respondents

Factors	B	S.E	Wald	df	Sig	Exp(B)	95% C.I. for EXP(B)	
							Lower	Upper
Constant	-2.350	0.881	7.112	1	0.008^a^	0.095		
Age (18–39 years old)	1.835	0.592	9.605	1	0.002^a^	6.266	1.963	20.002
Age (40–59 years old) ^a^Age (> 59 years old) (Ref)	0.878	0.618	2.018	1	0.155	2.405	0.717	8.076
Ethnic group Jakun ^a^Others (Ref)	0.570	0.682	0.697	1	0.404	1.768	0.464	6.733
No religion ^a^With religion (Ref)	0.482	0.395	1.486	1	0.223	1.619	0.746	3.512
Presence of woodstove in the house Yes ^a^No (Ref)	1.090	0.381	8.176	1	0.004*	2.975	1.409	6.283

*S.E.* Standard error, *df* Degree of freedom, *Sig* Significance, *C.I.* Confidence interval (dependent variable: FEV_1_: normal = 0, abnormal = 1; independent variable: Age: 18–39 years old = 0, 40–59 years old = 1, > 59 years old = 2; Ethnic group: Jakun = 0, Others = 1; Religion: No religion = 0, With religion = 1; Presence of woodstove in the house: Yes = 0, No = 1)

^a^Significant at *p* < 0.05

**Table 6:** Predictors of the Forced Vital Capacity (FVC) among respondents

Factors	B	S.E	Wald	df	Sig	Exp(B)	95% C.I. for EXP(B)	
							Lower	Upper
Constant	-1.310	0.885	2.193	1	0.139	0.270		
Age (18–39 years old)	1.269	0.645	3.873	1	0.049^a^	3.559	1.005	12.600
Age (40–59 years old) ^a^Age (> 59 years old) (Ref)	0.929	0.694	1.795	1	0.180	2.533	0.650	9.866
Ethnic group Jakun Others (Ref)	0.769	0.693	1.230	1	0.267	2.157	0.554	8.391
No religion With religion (Ref)	0.544	0.452	1.450	1	0.229	1.722	0.711	4.174
Presence of woodstove in the house Yes No (Ref)	0.986	0.435	5.128	1	0.024^a^	2.679	1.142	6.288

*S.E.* Standard error, *df* Degree of freedom, *Sig* Significance, *C.I.* Confidence interval (dependent variable: FVC: normal = 0, abnormal = 1; independent variable: Age: 18–39 years old = 0, 40–59 years old = 1, > 59 years old = 2; Ethnic group: Jakun = 0, Others = 1; Religion: No religion = 0, With religion = 1; Presence of woodstove in the house: Yes = 0, No = 1), Ref = reference

^a^Significant at p < 0.05.

**Table 7:** Predictors of the ratio of Forced Expiratory Volume in one second (FEV_1_) and Forced Vital Capacity (FVC) (FEV_1_/FVC) among respondents

Factors	B	S.E	Wald	df	Sig	Exp(B)	95% C.I. for EXP(B)	
							Lower	Upper
Constant	-1.946	0.756	6.626	1	0.010^a^	0.143		
No religion With religion (Ref)	-17.320	2656.243	0.000	1	0.995	< 0.001	< 0.001	-
Presence of woodstove in the house Yes No (Ref)	-17.102	2683.658	0.000	1	0.995	< 0.001	< 0.001	-

S.E.: Standard error; df: degree of freedom; Sig.: significance, C.I.: confidence interval (dependent variable: FEV_1_/FVC: normal = 0, abnormal = 1; independent variable: Religion: No religion = 0, With religion = 1; Presence of woodstove in the house: Yes = 0, No = 1). Ref = reference

^a﻿^Significant at *p* < 0.05

## Discussion

This study highlights the lung function status of the Orang Asli community in Tasik Chini and the associated factors. The study findings also add to the existing literature about the respiratory health of an indigenous community in a tropical region. Most of the respondents who were involved in this study were Orang Asli community aged 18 years old and above and lived in the five villages in Tasik Chini, Pahang. More than half of the respondents had no history of having respiratory problems during their lifetime and never sought any treatment in the clinics and hospitals due to any health problem especially related to respiratory problem. This study was the first experience for them to be introduced with one of the methods to measure lung functions, which is spirometry. The data collected in this study include sociodemographic factors, the house conditions and fuel that the respondents used frequently during cooking, smoking habits and other factors related to indoor environment. The finding on more than half of the respondents had no history of having respiratory problems during their lifetime contradicts some studies carried out among indigenous communities in other countries. Almost 20% of Australian indigenous patients were reported with multiple presentations of respiratory disorders to emergency department compared to 1% of non-indigenous patients [41]. A review of selected respiratory diseases (asthma, upper and lower respiratory conditions) among Aboriginal and Torres Strait Islander children showed that they were in the top ten specific conditions responsible for the total burden of diseases [42]. The lower respiratory problem among the Orang Asli community in this study should further be investigated.

### Sociodemographic characteristics of respondents

From a total of 211 respondents, the 18–39 years old group was the highest group who participated in this study with nearly equal number of male and female involved. Most of the respondents were from the Jakun tribe. The findings showed that there were significant associations between FEV_1_ and age, ethnic group, religion, and presence of woodstove in their house; between FVC and ethnic group, religion, type of house (traditional and modern) and presence of woodstove in their house; and between FEV_1_/FVC ratio and ethnic group and presence of woodstove in their house.

### Age

The respondents aged 18–39 years old have abnormal FEV_1_ level compared to older respondents. A cross-sectional study measuring lung function abnormalities among Australian indigenous community reported that despite the relatively young age (mean = 49 years, SD = 12.9 years), their lung function was generally low, in which the mean % predicted values were FEV1 = 55% (SD = 20.5%) and FVC = 61% (SD = 15.6%) [42]. However, this finding contradicted the finding of research carried out by Thomas et al. (2019) [43] who reported that lung functions (FEV_1_, FVC and PEFR) declined with age among individuals without known lung disease. FEV_1_ is declining with age and showed further declining rate after 70 years old. Air space size increased with age due to loss of supporting tissue. Reduction in chest wall compliance and increment in air tapping are also associated with aging. Even though these changes developed as people aged, the respiratory system has the ability to maintain adequate oxygenation and ventilation through the entire life span [44]. Thus, several studies had proven that as people become older, their lung functions decline [43–46]. Despite these changes, the respiratory system can maintain adequate oxygenation and ventilation during the entire life span.

As the decline in lung functions is a physiological change in ageing, several interventional studies had been carried out to improve the chest wall compliance before the starting of age-related musculoskeletal changes that have an impact on lung function improvement [44, 47–50]. Thus, it may become one of the possible interventions to slow down the declining process of lung functions as ageing progress if the exercise was done from early age.

### Ethnicity

Majority of the residents in Tasik Chini settlement are from the Jakun tribe and more than 80% of them have no religion (animists). The results from the analysis showed that the ethnicity (Jakun) and religion (no religion) have lower FEV_1_ and FVC levels compared to other ethnicities and those who have religion.

There are very limited studies on lung functions among Orang Asli community in Malaysia. The predicted value for lung function parameters also cannot be found during the process of interpretation of the spirometry data according to the proper predicted value that fits Orang Asli community. This reason can also be explained from the results of lung function parameters that we obtained. The number of respondents with abnormal FEV_1_ and FVC was very high, which is more than 75% from the total number of respondents who participated in our study. Several previous studies found that there was ethnic difference in lung functions [51–54]. One of the studies conducted on ventilator function and its relation to ethnicity among West London population showed that there was a clear difference in FEV_1_ and FVC between the three studied ethnicities (white, black, and mixed), while FEV_1_/FVC ratio remain the same. FEV_1_ and FVC was lower in black women and men, compared to white and mixed ethnic [54]. It takes into account that different ethnic has different anthropometric measures, providing different lung function levels. Thus, there is a connection between ethnicity and lung functions.

### Type of house and effect on lung functions

The type of house where respondents lived has two categories (modern and traditional) that have an association with FVC level. Respondents who lived in traditional houses have a low FVC level compared to those who lived in modern houses. This finding is also related to the exposure to solid fuel (woodstove) as the respondents who lived in traditional houses widely used woodstove rather than cleaner fuel such as LPG.

Different structures and construction materials from different types of housing may affect household indoor environment and housing conditions. People living inside different types of houses may be exposed to different levels of indoor pollutants, leading to different respiratory health outcomes. A study using samples of US adults from the year 1999–2006 National Health and Nutrition Examination Survey (NHANES) had been conducted to find out different respiratory health outcomes among the people who lived in different types of housing (townhouses, apartments, and mobile homes). The study found that people living in mobile homes had worse respiratory health outcomes compared to people living in townhouses and apartments [55].

Several studies investigate the relationship between lung functions and housing conditions and rural and urban living among children. A study by Kuti et al. (2017) [56] among children in Nigeria found that FEV_1_ and FVC were higher among male urban children compared to rural area. They explained that the results they received were due to children who lived in rural areas being exposed to more indoor air pollutants as they use unclean fuels for cooking and live in overcrowded houses. Other similar study that was conducted among Wuhan population in China showed that children who lived in urban area have a higher FVC compared to urban population. Thus, the findings supported the previous study [57].

In contrary, from the above research, a cross sectional study to compare whether rural and urban children may have different lung functions and respiratory health problem was done in Nigeria. The study found that there was no difference in lung functions among Nigerian children and adolescents living in either rural or urban areas [58].

### Exposure to coal combustion from woodstove

The findings of this study showed that FEV_1_ levels were significantly associated with age group (18–39 years old) and presence of woodstove in the house. FVC levels were significantly associated with the presence of woodstove in the house, but no significant association was observed between all predictors and FEV_1_/FVC ratio. The respondents who lived in the modern house and have woodstove in their house have abnormal FVC level compared to the respondents who lived in the traditional type of house and who did not have woodstove in their house. The findings correspond to previous research that was done on population in Northwest Ethiopia where wood fire use (AOR = 0.37 at 95% CI: 0.16–0.85) and living in mud- and wood-walled houses (AOR = 0.53 at CI: 0.32–0.89) were significantly associated with respiratory symptoms and impaired lung functions among respondents [59].

Several previous research stated that charcoal was a better fuel compared to open fire and firewood in view of coal produces less particulate matter than open fire and wood [60, 61], but that research contradict the research that was done in rural and peri urban Sierra Leone in 2011. The findings showed that kitchens of the house using wood stove has significantly higher suspended particulate matter compared to the kitchen that use charcoal stoves [62].

Other study that was conducted among 250 children in Ilesa, Nigeria found that those children who were exposed to unclean fuel from household cooking had significantly lower lung functions compared to children who lived in the urban areas and use clean fuel for cooking [56]. There is consistent evidence from a study done by Barone-Adesi et al. (2012) [63] that reported women who were exposed to coal smoke in their house had increased risk of lung cancer. The deterioration of lung functions among solid fuel users, especially coal and wood users, is due to the concentration of particulate matter and other toxic gases emitted during coal combustion. Pollutants from indoor coal combustion include the mixture and combination of gases and aerosols such as PM, CO, SO2 and others [40].

The use of cleaner fuel such as gas and LPG may reduce the risk of impaired lung functions. The finding is supported by a study that was conducted to examine the impact of a clean fuel which is LPG on respiratory outcomes among female respondents in two villages in India who used two different kinds of fuel (biomass and LPG). The study found that the use of a clean fuel cookstove which is LPG, improved lung function. Whereas women who primarily cook with dung biomass smoke had a reduction in percent predicted FEV_1_/FVC as compared to women who cook with LPG [64].

There are very limited intervention studies to modify the adverse respiratory outcomes from solid fuel smoke exposure. One of the most effective ways of eliminating those exposure is to change from solid fuel to cleaner fuels such as electricity and LPG. It is also suggested that if still need to use solid fuel, outdoor cooking is recommended as it will reduce the produced smoke from directly entering their house. The other way to reduce the exposure is by building up the partition between kitchen and living room. The presence of partition to the kitchen does not reduce the exposure of the cook but reduces exposure to other members in the household [65]. Outcomes from interventions, such as better stove design and maintenance, have not been adequately studied in developed countries.

### Exposure to environmental tobacco smoke (ETS)

According to USEPA, ETS is a human lung carcinogen that is responsible for approximately 3,000 lung cancer deaths among non-smoker in U.S. every year as they carry and deliver over 4,000 compounds including carcinogenic agents such as benzo(a)pyrenes, PAHs, and tobacco-specific nitrosamines [66, 67]. Infants and young children are regarded as a vulnerable group that is very sensitive to ETS. Exposure to ETS may cause development of new cases of asthma as the exposure leads to lung function impairment [66].

In our study, ETS (active and passive smoking) exposure was associated with decline in lung function. More than half of the respondents were current smokers (53.1%) and 41.7% were non-smokers. Of 53.1% current smokers, 30.4% of them smoked inside their house. This will increase the risk of exposure to ETS among their family members who lived in the same house.

The impact of tobacco on adult health requires urgent attention especially among women as several studies reported that women who were exposed to ETS had impaired lung functions. A study on environmental tobacco smoke exposure and pulmonary function among non-smoker male found that there was no evidence that ETS exposure was related to decreased lung functions. In contrary to adult female from the same study, ETS exposure is associated with decreased pulmonary function especially those with asthma [68]. Fewer studies have reported the association of exposure to ETS and declining lung functions among adult respondents and this often produces inconsistent results. Study by Skogstad et al. (2006) [69] described that the self-reported exposure to ETS was associated with decrease in lung function parameters (FEV_1_, FVC, FEV_1_/FVC ratio and PEFR). The other study that had been conducted on ventilatory function among Kuching, Sarawak population showed that smoking without the presence of respiratory symptoms was not associated with lung function declination [70].

Jakkola et al. (2019) [71] in their study reported the decline in lung functions especially FEV_1_ levels among regular smokers and former smokers with newly diagnostic asthma and it has a dose response pattern. The more the amount of smoking, the more obvious the decline in lung functions of the studied individuals. The results of two studies [72, 73] reported different findings from other studies that had been discussed because both studies were unable to link exposure to ETS with changes in lung functions in adults. Kuti et al. (2017) [56] in their study reported that there was no significant association between lung function parameters and exposure to passive smoking among children as there is a very low percentage of children who were exposed to passive tobacco smoke in their house. The relevance of timing of exposure in the associations of secondhand tobacco smoke (SHS), pets, and dampness or mold exposure with lung function is unclear. A study by Milanzi et al. (2020) [74] investigated the relevance of timing of exposures for lung function in adolescence. They found that there was a decline of FEV_1_ growth yearly since early childhood. Thus, it explained that exposure to SHS during childhood may lead to the reduction of lung function growth and lower attained lung function.

### Exposure to residential mold or dampness

In this study, more than half of the respondents (52.6%) answered that there was a wet/damp stain in their house. As we know, dampness area in the house is the favored condition for the mold to grow and later may affect respiratory system [75]. A population-based study among 269 non-asthmatic adults from South Finland found that FEV_1_ and FVC level was reduced on exposure to mold odor especially among women [76]. A recent study among England population showed that the respondents who were exposed to visible mold and moldy odor have a higher risk to develop asthma especially among adults aged 50 years old (odds ratio (OR) 2.4, 95% confidence interval (CI) 1.10–5.34) and the risk was higher among female than in male (OR 3.5, 95% CI 1.37–9.08). According to this study, exposure to visible mold growth and moldy odor are not the risk factors for wheeze or allergy [77].

Other studies also supported this finding to find the relationship between reduced lung functions caused by dampness and mold with gender. Here, the study reported that women who live in the house with dampness had reduced FEV_1_ of 2.25 ml/year (95% CI 4.25 to 0.25). The bedroom with observed damp spot was associated with a significant reduction in FEV_1_ of 7.43 ml/year (95% CI 13.11 to 1.74) [78]. Caillaud et al. (2018) [79] identified 61 publications that discussed the relationship between lung functions and presence of mold and dampness in the house. The presence of mold and mold odor were related to exacerbations of asthma in children as evidence of a causal relationship. Whereas, if it happens at workplace, it will provoke the development of occupational asthma. This systematic review also reported the association between exposure to mold and allergic rhinitis with sufficient evidence of association.

### Lung function test findings

From this study, we found that all lung function parameters were lower than predictive values. This finding is consistent with the only research on lung functions among Malaysian aborigines conducted in 1971 that found all parameters of spirometer including FEV_1_, FVC, PEFR were lower than predicted values [80]. However, this study did not specify the ethnicity of the Orang Asli population studied. The lower than predicted value may be due to the short/small stature of the respondents. Bhatti et al. (2014) [81] explained that tall stature has higher static lung volumes and capacities compared to the person with short stature.

In contrary to the study that was conducted on PEFR among Orang Asli Semai who lived in Cameron Highland, more than half of the respondents had PEFR that reach 80% of their predicted value. The findings might be different due to the altitude where the Orang Asli community lived [37]. The higher the altitude, the higher the lung volume, resulting in better lung function [82, 83]. The present findings might not be enough to determine whether the decline in all parameters studied compared to the predictive values was due to physiological differences or pathological changes. More studies can be done to explore further.

## Limitations

There are some limitations in this study that need to be acknowledged. First, the use of cross-sectional design limits causal inference. Therefore, past exposure to household environmental factors and recent changes in household environmental exposure were not assessed. Second, most of the respondents’ information was self-reported. All information such as sociodemographic characteristics, type and condition of house, smoking habit, and exposure to environmental tobacco smoke (ETS), health status factor, smoke exposure factor, home environment factor and dust factor were subjected to recall bias, misclassification and incomplete information.

## Conclusions

From this study, it can be concluded that the lung function parameters (FEV_1_ and FVC) of the Orang Asli community in Tasik Chini, Pahang were lower than the predictive value, whereas FEV_1_/ FVC (%) ratio and PEFR were within the predictive value. The findings showed that FEV_1_ levels were significantly associated with age group (18–39 years old) (*p* = 0.002) and presence of woodstove in the house (*p* = 0.004). FVC levels were significantly associated with presence of woodstove in the house (*p* = 0.004), whereas there were no significant associations between all factors and FEV_1_/FVC levels. Thus, environmental interventions such as replacing the use of woodstove with LPG, need to be carried out to prevent further worsening of respiratory health among Orang Asli who lived far from health facilities. Moreover, closer health monitoring is crucial especially among the younger and productive age group.

### Supplementary Information

Supplementary Material 1.

## Data Availability

All data generated or analysed during this study are included in this published article.
