# Supplementary material for: Factors related with lung functions among Orang Asli in Tasik Chini, Malaysia: a cross-sectional study
Source: BMC Public Health. 2024 Jul 5;24:1791. doi: 10.1186/s12889-024-19296-x (PMC11225401; doi:10.1186/s12889-024-19296-x)
Supplement: Supplementary file 1 — Supplementary Material 1. [file 12889_2024_19296_MOESM1_ESM.docx]

STROBE guidelines checklist.

| Item number | Section/topic | Recommendation | Feedback/Corrections |
| --- | --- | --- | --- |
| 1 | Title and abstract | Indicate the study’s design with a commonly used term in the title or the abstract.  Provide in the abstract an informative and balanced summary of what was done and what was found. | The study’s design has been stated in the title and abstract.  The summary of what was done and what was found in the study has been provided in the abstract. |
|  | Introduction |  |  |
| 2 | Background/rationale | Explain the scientific background and rationale for the investigation being reported. | The scientific background and rationale for the investigation has been reported in the background section. |
| 3 | Objectives | State specific objectives, including any prespecified hypotheses. | The specific objectives and hypotheses have been stated. |
|  | Methods |  |  |
| 4 | Study design | Present key elements of study design early in the manuscript. | The study design has been described in the methodology section of the manuscript. |
| 5 | Setting | Describe the setting, locations, and relevant dates, including periods of recruitment, exposure, follow‑up, and data collection. | Information on dates of data collection has been added in the manuscript. |
| 6 | Participants | Cross‑sectional study – give the eligibility criteria, and the sources and methods of selection of participants. | The eligibility criteria and methods of respondents’ selection has been described. |
| 7 | Variables | Clearly define all outcomes, exposures, predictors, potential confounders, and effect modifiers; give diagnostic criteria, if applicable. | All variables in the study have been clearly defined. |
| 8 | Data sources/measurement | For each variable of interest, give sources of data and details of methods of assessment (measurement); describe comparability of assessment methods if there is more than one group. | Sources of data and details of methods of assessment have been described in the methodology. |
| 9 | Bias | Describe any efforts to address potential sources of bias. | Potential sources of bias were reported in the study limitation section. |
| 10 | Study size | Explain how the study size was arrived at. | Sample size was calculated based on Krejcie & Morgan (1970). |
| 11 | Quantitative variables | Explain how quantitative variables were handled in the analyses; if applicable, describe which groupings were chosen and why. | Data analysis of quantitative variables and comparison between groups was stated in the data analysis section. |
| 12 | Statistical methods | Describe all statistical methods, including those used to control for confounding.  Describe any methods used to examine subgroups and interactions.  Explain how missing data were addressed.  Cross‑sectional study – if applicable, describe analytical methods taking account of sampling strategy.  Describe any sensitivity analyses. | Data analysis of quantitative variables and comparison between groups was stated in the data analysis section. There were no missing data. |
|  | Results |  |  |
| 13 | Participants | Report numbers of individuals at each stage of study – e.g., numbers potentially eligible, examined for eligibility, confirmed eligible, included in the study, completing follow‑up, and analyzed.  Give reasons for nonparticipation at each stage. Consider use of a flow diagram. | The total number of respondents recruited in this study was 211. |
| 14 | Descriptive data | Give characteristics of study participants (e.g., demographic, clinical, social) and information on exposures and potential confounders.  Indicate number of participants with missing data for each variable of interest. | The sociodemographic characteristics of the respondents are presented in Table 1, while the descriptive results of the dependent variables are presented in Table 2. There were no missing data. |
| 15 | Outcome data | Cross‑sectional study – report numbers of outcome events or summary measures. | Number of outcome events has been reported. |
| 16 | Main results | Give unadjusted estimates and, if applicable, confounder‑adjusted estimates and their precision (e.g., 95% confidence interval); make clear which confounders were adjusted for and why they were included.  Report category boundaries when continuous variables were categorized.  If relevant, consider translating estimates of relative risk into absolute risk for a meaningful time period. | The main results were presented accordingly starting from descriptive data, bivariate analysis, and lastly multivariate analysis, where the confounders were adjusted. |
| 17 | Other analyses | Report other analyses done – e.g., analyses of subgroups and interactions, and sensitivity analyses. | Each lung function parameter (dependent variable) was categorized into two – normal and abnormal. All the bivariate and multivariate analyses were carried out to predict the abnormal lung function status. |
|  | Discussion |  |  |
| 18 | Key results | Summarize key results with reference to study objectives. | All key results were summarized according to study objectives. |
| 19 | Limitations | Discuss limitations of the study, taking into account sources of potential bias or imprecision; discuss both direction and magnitude of any potential bias. | Limitations of the study were discussed in the study limitation section in the manuscript. |
| 20 | Interpretation | Give a cautious overall interpretation of results considering objectives, limitations, multiplicity of analyses, results from similar studies, and other relevant evidence. | Results were interpreted accordingly and were compared with previous studies. |
| 21 | Generalizability | Discuss the generalizability (external validity) of the study results. | The study results and intervention approach were generalized for other Orang Asli community. |
|  | Other information |  |  |
| 22 | Funding | Give the source of funding and the role of the funders for the present study and, if applicable, for the original study on which the present article is based. | This study was funded by the PPUKM Fundamental Grant (FF-2017-471). |
